# Timing Is Important—Management of Metabolic Syndrome According to the Circadian Rhythm

**DOI:** 10.3390/biomedicines11041171

**Published:** 2023-04-13

**Authors:** Ksenija Zečević, Nataša Popović, Aleksandra Vuksanović Božarić, Mihailo Vukmirović, Manfredi Rizzo, Emir Muzurović

**Affiliations:** 1 Faculty of Medicine, University of Montenegro, 81000 Podgorica, Montenegro; 2 Cardiology Clinic, Clinical Center of Montenegro, 81000 Podgorica, Montenegro; 3 Promise Department, School of Medicine, University of Palermo, 90127 Palermo, Italy; 4 Department of Internal Medicine, Endocrinology Section, Clinical Center of Montenegro, 81000 Podgorica, Montenegro

**Keywords:** circadian clocks, metabolic syndrome, diet, exercise therapy, chronotherapy, peroxisome proliferator-activated receptors

## Abstract

Physiological processes occur in accordance with a rhythm regulated by the endogenous biological clock. This clock is programmed at the molecular level and synchronized with the daily light–dark cycle, as well as activities such as feeding, exercise, and social interactions. It consists of the core clock genes, Circadian Locomotor Output Cycles Protein Kaput (CLOCK) and Brain and Muscle Arnt-Like protein 1 (BMAL1), and their products, the period (PER) and cryptochrome (CRY) proteins, as well as an interlocked feedback loop which includes reverse-strand avian erythroblastic leukemia (ERBA) oncogene receptors (REV-ERBs) and retinoic acid-related orphan receptors (RORs). These genes are involved in the regulation of metabolic pathways and hormone release. Therefore, circadian rhythm disruption leads to development of metabolic syndrome (MetS). MetS refers to a cluster of risk factors (RFs), which are not only associated with the development of cardiovascular (CV) disease (CVD), but also with increased all-cause mortality. In this review, we consider the importance of the circadian rhythm in the regulation of metabolic processes, the significance of circadian misalignment in the pathogenesis of MetS, and the management of MetS in relation to the cellular molecular clock.

## 1. Introduction

Metabolic syndrome (MetS) refers to a cluster of risk factors (RFs) which are not only associated with the development of cardiovascular (CV) disease (CVD), but also with increased all-cause mortality [1]. In 2009, several societies harmonized the definition of MetS and agreed that the diagnosis of MetS should be made on the basis of three abnormal findings out of five components: elevated fasting plasma glucose (FPG) level, i.e., insulin resistance (IR); increased waist circumference (WC) i.e., central obesity; elevated triglycerides (TGs); reduced high-density lipoprotein cholesterol (HDL-C) levels; and elevated blood pressure (BP) [2].

The prevalence of MetS has demonstrated continuous growth globally and was estimated to be around 20–25% in 2021 [3]. In addition to an increased risk for CVD, MetS is associated with a greater risk of type 2 diabetes mellitus (T2DM), nonalcoholic fatty liver disease (NAFLD), polycystic ovary syndrome, gout, dementia, and some types of cancer [4,5]. The etiology of MetS is multifactorial, and current knowledge implies that MetS develops as a result of environmental RFs combined with genetic and epigenetic factors [1]. In addition to the well-known environmental factors, such as increased daily caloric intake and physical inactivity, recent Nobel prize-winning research by Jeffrey C. Hall, Michael Rosbash, and Michael W. Young has shed light on the molecular mechanisms responsible for the connection between circadian misalignment and the pathogenesis of MetS [6,7]. They identified a set of genes with rhythmic diurnal expression, which are involved in the regulation of metabolic pathways and hormone release, but also in the regulation of many other physiological processes [6,7]. Internal desynchrony in the organism, with out-of-phase expression of circadian genes in different tissues, has been associated with metabolic disorders [8,9].

The main goal of this review is to clarify the importance of the circadian rhythm in the regulation of metabolic processes. The significance of circadian misalignment in the pathogenesis of MetS and the implications of this misalignment in the management of patients with MetS are also considered.

## 2. The Link between Circadian Clock and Metabolism

Circadian rhythms are physiological, mental, and behavioral changes that follow a 24 h cycle [10]. Even though cells are able to generate rhythms autonomously, the suprachiasmatic nuclei (SCN) of the hypothalamus act as principal circadian pacemakers and synchronize rhythms in all other tissues, in accordance with environmental cues such as light exposure, feeding patterns, exercise, and weather conditions [10].

### 2.1. Core Clock Genes

**Figure 1:**
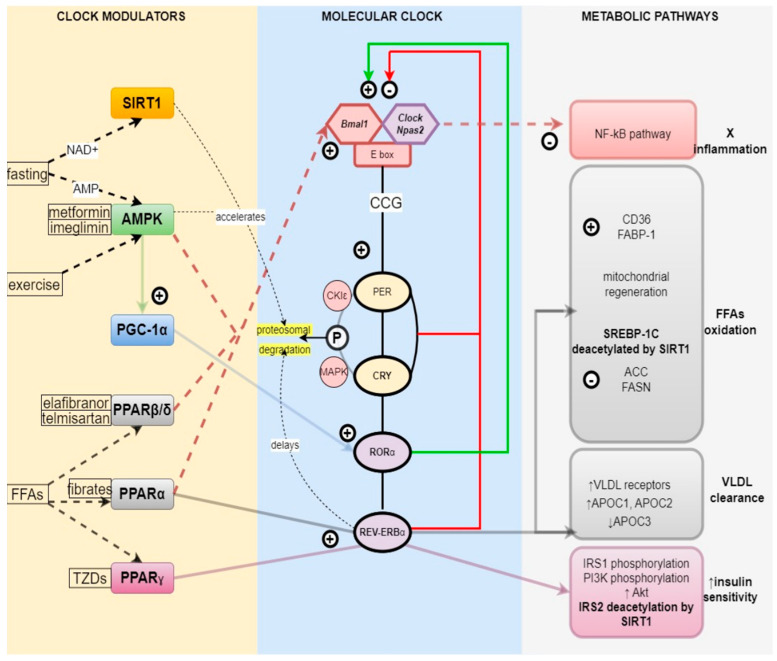
Relationship between molecular clock and metabolism. SIRT1 senses nutrient deprivation through elevated NAD+ levels and accelerates PER degradation in proteosomes, phase−advancing the molecular clock. SIRT1 also deacetylates SREBP−1C and IRS2, increasing FFA oxidation and insulin sensitivity, respectively. AMPK is upregulated by fasting through high AMP levels, exercise, and medications such as metformin and imeglimin. AMPK accelerates CRY degradation, resetting the molecular clock. It downregulates the NF−kB pathway, thereby reducing inflammation. It also promotes PGC−1α expression, which, through the RORα clock component, stimulates mitochondrial regeneration and FFA oxidation. PPARβ/δ and PPARα inhibit the NF−kB pathway and reduce inflammation, as well as increase FFA oxidation. PPARα stimulates REV−ERBα-mediated VLDL clearance. PPARγ increases insulin sensitivity via REV−ERBα-mediated pathway. Abbreviations: SIRT1, sirtuin1; AMPK, adenosine monophosphate (AMP) activated protein kinase; PGC−1α, peroxisome proliferator−activated receptor−gamma coactivator 1α; NAD+, nicotinamide adenine dinucleotide; AMP, adenosine monophosphate; PPARβ/δ, proliferator-activated receptor β/δ; PPARα, proliferator−activated receptor α; PPARγ, peroxisome proliferator−activated receptor γ; FFA(s), free fatty acid(s); TZDs, thiazolidinediones; Bmal1, basic helix−loop−helix ARNT−like 1; Clock, Circadian Locomotor Output Cycles Protein Kaput; Npas2, neuronal PAS domain protein 2; CCG, clock-controlled genes; PER, period proteins; CRY, cryptochrome proteins, RORα, retinoic acid-related orphan receptor α; REV−ERBα, reverse-strand avian erythroblastic leukemia (ERBA) oncogene receptor α; MAPK, mitogen−activated protein kinase; CK1ε, casein kinase I isoform epsilon; NF−kB, nuclear factor kappa−light−chain−enhancer of activated B cells; FABP−1, fatty acid binding protein 1; SREBP−1C, sterol regulatory element-binding protein 1; ACC, acetyl−CoA carboxylase; FASN, fatty acid synthase; VLDL, very low density lipoprotein; APOC, genes encoding apolipoproteins C; IRS, insulin receptor substrate; PI3K, phosphatidylinositol−3−kinases; Akt, protein kinase B.

Circadian rhythms within the cells are programmed by the molecular clock, which is composed of a group of core clock genes [10]. The transcription of these genes is regulated through a negative feedback mechanism [10]. During the light phase of the day, Brain and Muscle Arnt-Like protein 1 (BMAL1) and Circadian Locomotor Output Cycles Protein Kaput (CLOCK), or its paralog neuronal PAS domain protein 2 (NPAS2), form a heterodimer and bind to E boxes in the promoters of various clock-controlled genes to promote their expression [10]. They also promote the synthesis of the circadian repressors, period (PER) and cryptochrome (CRY), which, upon accumulation, enter the nucleus and inhibit the activity of clock machinery [10]. This PER- and CRY-mediated suppression of clock gene transcription occurs during the dark phase of the day [10]. *PER* gene expression is controlled at the mRNA translational level [11]. The mitogen-activated protein kinases (MAPKs) interacting protein kinases (MNKs) phosphorylate the cap-binding protein eukaryotic translation initiation, factor 4E (eIF4E), and promote PER1 and PER2 mRNA translation [11]. PER and CRY proteins are also regulated by post-translational modifications and ubiquitination [11]. PER proteins are phosphorylated by casein kinase (CSNK) 1δ and ε, and dephosphorylated by protein phosphatase 1(PP1) [12]. CRY proteins are phosphorylated by adenosine monophosphate (AMP) activated protein kinase (AMPK) [11]. Phosphorylated PER and CRY proteins are degraded by ubiquitination in the proteosomes, and a new cycle begins [10] (Figure 1).

### 2.2. Reverse-Strand Avian Erythroblastic Leukemia (ERBA) Oncogene Receptors (REV-ERBs) and Retinoic Acid-Related Orphan Receptors (RORs)

An interlocked feedback loop involving the REV-ERB and ROR nuclear receptor family fine-tunes core clock function [13,14].

*REV-ERBα* expression is controlled by core clock genes [13]. On the one hand, CLOCK/BMAL1 activates *REV-ERBα* transcription, via E box DNA response elements found within the *REV-ERBα* promoter, and on the other hand, PER/CRY transrepresses *REV-ERBα* transcription [13]. This results in circadian fluctuations of REV-ERB*α* [13]. In turn, accumulated REV-ERB*α* represses *BMAL1* transcription passively, through binding on two DNA response elements that are located in the *BMAL1* promoter, or actively, by recruiting the nuclear receptor corepressor- histone deacetylases 3 (NCoR-HDAC3) complex [14]. REV-ERBs are not necessary for core clock rhythmicity, but have a major role in stabilizing the circadian pacemaker period [15]. REV-ERBα delays PER phosphorylation and extends the circadian period, by suppressing *Ppp1r1b*, which encodes the PP1 inhibitor dopamine- and cyclic-AMP-regulated phosphoprotein of molecular weight 32,000 (DARPP-32) [15]. *REV-ERBα*-deficient mice are not arrhythmic, but their free-running period is shortened to 21 h [15]. REV-ERB*α* is implicated in metabolism through activation by both endogenous ligands, such as hem, as well as synthetic ligands, such as SR9009 and SR9011 [16]. REV-ERBα activation with ligands SR9009 and SR9011 in mice decreased the expression of lipogenic enzymes (fatty acid synthase [*Fasn*] and Stearoyl-CoA desaturase [*Scd*]) and cholesterologenic regulator proteins (3-hydroxy-3-methylglutaryl-CoA reductase [*Hmgcr*] and Sterol Regulatory Element-Binding Transcription Factor 2 [*Srebf2*].

REV-ERBα activation induced the expression of genes whose products are involved in free fatty acid (FFA) and glucose oxidation pathways, such as carnitine palmitoyltransferase 1b [*Cpt1b*], PPARγ coactivator 1-beta [Pgc-1β], M2 isoform of pyruvate kinase muscle [*Pkm2*], and hexokinase 1 [*Hk1*]) [16]. Apolipoprotein C-III, a component of very low density lipoproteins (VLDLs), is downregulated by REV-ERBα, and mice lacking REV-ERB exhibit hypertriglyceridemia [17] (Figure 1). According to research by Lazar and colleagues, in vitro gluconeogenic Pepck gene repression in human hepatoma HepG2 cells increased as a result of heme binding to REV-ERBα [18].

REV-ERB rhythmicity also influences appetite. Deletion of *REV-ERBs* is associated with decreased leptin sensitivity and increased appetite [15].

Another nuclear receptor, RORα, competes with REV-ERBα for the *BMAL1* binding site [13]. In contrast to REV-ERBα, which is a *BMAL1* repressor, RORα is a *BMAL1* activator [13]. Hence, RORα and REV-ERBα, expressed 12 h out of phase with one another, work together as a regulatory loop, alternately activating and repressing *BMAL1* in an oscillatory manner [13,15].

Rorα enhances activity of the human Apolipoprotein C-III (Apo C-III) gene (*APOC3)* [19]. Apo C-III overexpression is characterized by hypertriglyceridemia and contributes to atherosclerosis [19].

### 2.3. Regulation of the Metabolism by the Circadian Clock

#### 2.3.1. Nutrient Sensors and the Clock—PPARα, β, γ

Peroxisome proliferator-activated receptors (PPARs) are members of the nuclear hormone receptor superfamily of ligand-activated transcription factors [20]. The three PPARs (α, δ, γ) have been shown to regulate carbohydrate, lipid, lipoprotein, and energy metabolism in a clock-controlled manner [20].

##### PPARα

PPARα directly positively regulates *Bmal1* through binding to peroxisome proliferator response element (PPRE), located at the position −1519 in the *Bmal1* promoter [21]. BMAL1, then, imposes a positive circadian regulation on PPARα transcription [21]. PPARα also increases *REV-ERBα* expression in human liver cells [17].

Natural ligands of PPAR*α* include a variety of FFAs and their derivatives, including acyl-coenzyme As (CoAs), oxidized FFAs, and eicosanoids [22]. The most important class of synthetic PPAR*α* ligands is that of fibrates, including gemfibrozil, bezafibrate, clofibrate, and fenofibrate, used in the treatment of dyslipidemia primarily associated with T2DM [22]. 

When activated, PPARα suppresses VLDL production by promoting FFA oxidation in the liver and downregulating the *APOC3* gene [23]. PPARα upregulates the expression of some genes for mitochondrial FFA oxidation, such as medium-chain acyl-CoA dehydrogenase (MCAD) or long-chain acyl-CoA dehydrogenase (LCAD) [24]. PPARα downregulates *APOC3* gene expression through REV-ERBα, which binds to a RevRE site located in the *APOC3* gene promoter adjacent to the TATA box [23]. In addition to suppressing VLDL production, PPAR*α* agonists stimulate clearance of VLDLs, mediated by lipoprotein lipase (LPL) in the liver [22]. As mentioned, PPAR*α* activation downregulates *APOC3*, which is an LPL inhibitor [22]. Furthermore, PPAR*α* stimulates hepatic expression of the VLDL receptor (Vldlr) [22]. PPAR*α* agonists raise plasma HDL levels in humans, which is most likely achieved via mRNA induction of apolipoprotein A-I (Apoa1) and A-II (Apoa2) [22]. The highest PPARα expression coincides with the beginning of the rest phase, which corresponds with high β-oxidation rates at rest [25].

PPARα decreases TG and FFA overload by upregulating the expression of genes that mediate TG hydrolysis, FFA transport, and β-oxidation in the liver, skeletal muscle, and adipose tissue [26]. Thereby, PPARα improves insulin sensitivity [26].

PPARα downregulates nuclear factor kappa-light-chain-enhancer of activated B cells (NF-κB) expression in a ligand-dependent trans-repression manner [26]. Expression of NF-κB is, on the other hand, upregulated in IR, and its activation initiates the inflammatory cascade [27]. Activation of NF-κB kinase-β (IKK-β) leads to expression of proinflammatory cytokines, such as tumor necrosis factor-alpha (TNF-α) and interleukin 6 (IL-6) [27]. TNF-α and IL-6 contribute to the development of IR and NAFLD [27]. TNF promotes IR and NAFLD by increasing adipocyte lipolysis and FFA levels, enhancing insulin receptor substrate-1 (IRS-1) phosphorylation, and suppressing AMPK activity [27]. IL-6 suppresses IL-1-induced secretion of insulin, by activating the c-Jun N-terminal kinase (JNK) pathway [27]. When activated by ligands, PPARα negatively regulates these proinflammatory pathways and can counteract NAFLD development [26].

##### PPARγ

PPARγ is mainly expressed in white adipose tissue (WAT), but also in the liver, kidneys, endothelium, and immune system [28]. It regulates adipogenesis, lipid metabolism, insulin sensitivity, and blood pressure, presumably through REV-ERB𝛼 activation [28]. PPARγ expression in adipose tissue and skeletal muscle peaks at the beginning of the active phase [20]. Natural ligands of PPARγ include unsaturated FFAs and their derivates, and its most well-known synthetic ligands are thiazolidinediones (TZDs), used in treatment of T2DM [28].

On the one hand, PPARγ increases glucose and lipid uptake and glucose oxidation, and on the other hand, it decreases FFA concentration and IR [29]. Target genes directly regulated by PPARγ include LPL, oxidized low density lipoprotein (LDL) receptor 1, *Cd36*, fatty acid-binding protein4/adipocyte protein 2 (*Fabp4/aP2)*, monoacylglycerol O-acyltransferase 1 (*Mogat1)*, fat-specific protein 27 *(Fsp27)* [30], which all favor adipocyte uptake of circulating FFAs [29,30]. Furthermore, PPARγ upregulates phosphoenolpyruvate carboxykinase, glycerol kinase, and the glycerol transporter aquaporin 7, which promotes recycling rather than the export of intracellular FFAs [29]. PPARγ agonists induce the PPARγ coactivator-1a (PGC-1α), which promotes mitochondrial biogenesis, leading to an increase in FFA oxidation [29]. PPARγ agonists also have the ability to redistribute fat from visceral to subcutaneous depots, increase adiponectin, and reduce tissue necrosis factors (TNFs) [31].

PPARγ agonists decrease IR both directly, by acting on insulin receptors, and indirectly, by lowering the FFA pool within the cytoplasm [32]. TZDs increase IRS-1 tyrosine phosphorylation, IRS-1-associated phosphoinositide 3-kinase (PI3K), and Akt activity [32] (Figure 1).

Activation of PPARγ also attenuates vascular dysfunction [28,33]. PPARγ agonists suppress the M1 macrophage phenotype, inhibiting the expression of pro-inflammatory cytokines TNFα, interleukin-1β (IL-1β), and IL-6, slowing down the progression of atherosclerotic plaques [33]. PPARγ agonists suppress the angiotensin II-induced phosphatidylinositol 3-kinase and MAPK in vivo, hindering the renin–angiotensin–aldosterone system (RAAS) mediated increase in BP [33].

##### PPARδ/β

PPAR-β/δ is ubiquitously expressed in humans [20]. It is activated by saturated and unsaturated long-chain fatty acids, prostacyclin, as well as synthetic ligands such as elafibranor and telmisartan [20]. Moreover, *PPAR-β/δ* expression is upregulated specifically in skeletal muscle during fasting [28]. Studies also suggest that PPAR-β/δ and physical exercise are tightly related [34]. Endurance training of 6 weeks boosted PPAR-β/δ protein expression in the tibialis anterior muscle by up to 2.6-fold [34]. Moreover, in a study in mice, *PPAR-β/δ* was upregulated exclusively after exercise in the early active phase [35].

PPAR𝛽/𝛿 controls the temporal expression of hepatic lipogenic genes, including acetyl-CoA carboxylase 1 (ACC1), ACC2, fatty acid synthase (FAS), and stearoyl-CoA desaturase-1 (SCD1), thus affecting fatty acid metabolism in the liver and PPAR-β/δ activation, leading to increased levels of FFA oxidation [28]. PPAR𝛽/𝛿 also suppresses macrophage-derived inflammation, thus having a potential role in attenuating atherogenesis [25]. A strong anti-inflammatory effect is linked to the pharmacological activation of PPAR-β/δ in endothelial cells, potentially through the involvement of antioxidative genes and the release of nuclear corepressors [25]. Moreover, investigation of the role of PPAR-β/δ in the modulation of NF-κB-driven inflammatory response confirmed the anti-inflammatory activity of this isotype [28].

#### 2.3.2. Energy Sensors and the Clock—AMPK, SIRT1, PGC1α

Feeding patterns can regulate clock-controlled gene transcription [36]. Cellular metabolism can only be effectively controlled by the circadian clock if the clock machinery is able to determine the energy status of the cell [37]. Clock machinery senses the energy status of the cell through nutrient sensors, sirtuin 1 (SIRT1), AMPK, and PGC1α [9,38].

##### SIRT1

SIRT1, a nicotinamide adenine dinucleotide (NAD^+^) dependent deacetylase, is upregulated by nutrient deprivation, and it directly binds to CLOCK-BMAL1 and rhythmically deacetylates PER2, promoting its degradation [38]. Thus, SIRT1 resets the clock by accelerating the onset of the new cycle [38] (Figure 1).

SIRT1 plays a crucial metabolic role, and SIRT1 protein levels are increased upon starvation in the liver [39]. Cyclic adenosine monophosphate (cAMP) responsive element-binding protein (CREB) positively activates SIRT1 gene transcription during fasting [40]. Furthermore, caloric restriction, by increasing the content of NAD+, stimulates SIRT1 activity [38].

SIRT1 decreases hepatic TG levels by inhibiting lipogenesis and stimulating fatty acid β-oxidationSIRT1 [40]. SIRT1 inhibits hepatic FFA and TG synthesis by inhibiting a key lipogenic activator, SREBP-1c, and stimulates β-oxidation by targeting PPARα and its coactivator, PGC-1α [40]. SIRT1 deacetylates SREBP-1c at Lys-289 and Lys-309, and downregulates its transcriptional activity on its lipogenic target genes, such as FASN and ACC [40]. Via SREBP-1c deacetylation, SIRT1 promotes its ubiquitination and proteasomal degradation [40]. SIRT1 increases transcriptional activity of the coactivator PGC-1α by deacetylation [40]. It is not known whether PPARα is also a direct target of SIRT1 deacetylation during fasting, but deletion of SIRT1 results in impaired PPARα signaling and decreased expression of genes involved in β-oxidation, whereas overexpression of SIRT1 leads to increased PPARα transcriptional signaling [40]. SIRT1 also antagonizes the action of PPARγ on genes mediating fat storage, such as the *aP2* promoter, thereby blocking adipogenesis and promoting fat mobilization in starved mice [38].

SIRT1 promotes protein B kinase (Akt) activation and insulin signaling on several levels [38]. SIRT1 deacetylates IRS-2, enhancing the tyrosine phosphorylation of IRS-2 [38]. SIRT1 promotes Akt’s binding to phosphatidylinositol 3,4,5-trisphosphate (PIP3), which is necessary for Akt membrane localization and activation [38]. The transcription of PTPN1, a suppressor of the insulin signal transduction cascade, is repressed by SIRT1 [38]. SIRT1 promotes adenosine triphosphate (ATP) production in pancreatic β cells, which shuts down the potassium channels, resulting in the influx of calcium and the secretion of insulin [38].

SIRT1 suppresses inflammatory cytokine expression and reactive oxygen species (ROS) formation [39]. Through this process, SIRT1 impedes the inflammatory component of metabolic disease [39]. SIRT1 can reduce histone H3K9 acetylation in the promoters of IL-6 and TNFα, blocking their expression [39]. SIRT1 has been reported to increase cellular ability to remove ROS by superoxide dismutase (SOD) activation. SIRT1 also inhibits the NF-κB-signaling pathway directly, by deacetylating the p65 subunit of the NF-κB complex [39]. ROS incite inflammation by upregulating the expression of adhesion molecules and pro-inflammatory cytokines IL-1, IL-6, TNF-, and NF-kB [39]. Inflammation contributes to IR, hyperglycemia, and abnormalities in redox signaling in tissues, which all lead to the progression of atherosclerosis [39].

##### AMPK

During fasting, AMPK, an energy sensor of high AMP/adenosine triphosphate (ATP) ratio, destabilizes the circadian transcriptional repressors CRY1 and PER2 and accelerates their degradation [9]. AMPK phosphorylates CRY1 and Casein Kinase 1 (CK1) ε. Phosphorylation by AMPK leads to the ubiquitination and degradation of CRY1 [41]. AMPK-mediated activation of CK1ε leads to the degradation of PER2 [41]. This results in phase advance in circadian oscillations [41]. AMPK activation also leads to an increase in NAD^+^ levels and, consequently, upregulation of SIRT1 [37]. Thus, AMPK could also modulate circadian gene expression indirectly, through SIRT1 activation [37].

AMPK is implicated in lipid and glucose metabolism [41]. Transcription of clock-controlled genes promoted by AMPK results in the inhibition of ACC, the rate-limiting enzyme in FFA synthesis, as well as in the inhibition of the gluconeogenesis pathway in the liver, and increased insulin sensitivity in the peripheral tissues [41].

AMPK suppresses the expression of NF-κB by increasing the expression of SIRT1, thereby minimizing the inflammatory response in WAT [39].

AMPK is upregulated and activated by different treatment interventions, including intermittent fasting [9], exercise in the early hours of the active phase [42], and medications such as metformin and imeglimin [41].

##### PGC-1α

PGC-1α has been shown to play a crucial role in the response to fasting [43]. It enhances *CLOCK*, *BMAL1,* and *REV-ERBα* transcription through RORα transcriptional activity potentiation [17]. Fasting enhances PGC-1α expression [43]. PGC-1α then triggers the fasting-induced activation of the gluconeogenic pathway and fatty acid oxidation [9,43]. Mice deficient in PGC1α display hypoglycemia and hepatic steatosis [17]. PGC-1α also regulates the expression of mitochondrial antioxidant genes, including manganese superoxide dismutase, catalase, peroxiredoxin 3 and 5, uncoupling protein 2, thioredoxin 2, and thioredoxin reductase, and thus prevents oxidative injury and mitochondrial dysfunction [43].

PGC-1α activates mitochondria biogenesis and increases mitochondrial function [12]. PGC-1 α upregulates the expression of a number of genes involved in the mitochondrial FFA oxidation pathway and the tricarboxylic acid (TCA) cycle [12]. Long-chain and very-long-chain FFAs are likewise stimulated by PGC-1α in their peroxisomal oxidation [12]. The level of PGC-1α positively correlates with cells’ capacity to completely oxidize FFAs, which may reduce intramuscular lipid deposition and increase tissue insulin sensitivity [43,44]. PGC-1α activates the expression of insulin-sensitive glucose transporter type 4 (GLUT4) in skeletal muscle, preventing IR [43].

The PGC-1α-signaling pathway is upregulated during intermittent fasting, which explains why intermittent fasting may protect against ROS, IR, and obesity [9]. PGC-1α is also activated by medications such as metformin, in an AMPK-mediated manner, and PPARα and γ agonists, such as bezafibrate and rosiglitazone, respectively [44].

#### 2.3.3. Indirect Circadian Control of Metabolism by Hormone Secretion

In addition to circadian patterns of transcription factors and enzyme expression, the secretion of melatonin, insulin, glucagon, glucagon-like-peptide-1, cortisol, leptin, adiponectin, ghrelin, and RAAS is also coordinated by the molecular clock [45,46,47,48,49,50,51]. All of these hormones are major regulators of metabolism, and therefore represent an important link between the circadian rhythm and metabolic processes [45,46,47,48,49,50,51].

##### Insulin

Insulin secretion rate and serum insulin concentration change throughout a circadian rhythm, increasing from a nadir between midnight and 6 am and reaching a peak between noon and 6 pm [52]. Insulin sensitivity follows the same circadian pattern, at its lowest during the night and increasing during the day [52]. Melatonin promotes β cell regeneration; therefore, the cells’ response to postprandial hyperglycemia is the best in the morning [53].

The circadian rhythm of insulin secretion, tissue sensitivity to insulin, and glucose tolerance is significantly impaired in obese people and patients with T2DM [54]. Insulin sensitivity in these individuals is the lowest in the morning, which could explain the dawn phenomenon [55]. Changes in the circadian rhythm of insulin secretion are accompanied by changes in the expression of clock genes [56]. People with T2DM have reduced expression of *Cry2*, *Per2*, and *Per3* in pancreatic islet cells compared to those without T2DM [56]. *Per3* expression was also reduced in the pancreatic islets of individuals with T2DM when cultured under glucolipotoxic conditions for 48 h [56].

A number of epidemiological studies suggest that lifestyle factors that disrupt circadian rhythms contribute to IR and T2DM [57,58,59]. In a study by Kervezee and colleagues, three days of simulated night shifts resulted in a significant decrease in insulin sensitivity and increase in postprandial glycemia levels [57]. Forced desynchrony protocols with 28 h days showed a 6% increase in glucose serum levels during circadian misalignment (when eating and sleeping 12 h out of phase from habitual schedules), despite a 22% rise in insulin levels over the entire sleep–wake cycle [58]. The results indicated decreased glucose tolerance, possibly due to reduced insulin sensitivity [58]. According to cross-sectional data from the New Hoorn Study cohort, social jetlag greater than 2 h, which is a sleep and wake time discrepancy of 2 or more hours between work and free days, was associated with a 2-fold increased risk of T2DM and MetS in younger participants (<61 years) [59].

On the other hand, alignment of circadian rhythms with external stimuli improves insulin sensitivity and glucose tolerance [60,61,62,63,64]. Sleep duration is linearly correlated with better glycemic control [60]. Increasing sleep duration by one hour decreased HbA1c by 0.174% (1.4 mmol/mol) [60]. Other lifestyle interventions that realign circadian rhythms, such as time-restricted feeding (TRF) and exercise timing, also improve insulin sensitivity and glycemic control [61,62,63,64,65].

##### GLP-1

Glucagon-like peptide-1 (GLP-1) is a peptide hormone that increases insulin secretion and decreases glucagon secretion from the pancreas in a glucose-dependent manner [66]. It stimulates adenylate cyclase and raises cAMP levels via Gs [67]. GLP-1 then activates protein kinase A (PKA), an exchange protein directly activated by cAMP (EPAC), through cAMP-dependent pathways to inhibit ATP-regulated potassium channels, increase the activity of L-type voltage-gated calcium channels, and open nonspecific cation channels [67]. This leads to increased calcium influx, thereby enhancing calcium-induced insulin secretion [67]. GLP-1 enhances the cell sensitivity to glucose and inhibits ATP-regulated potassium channels, which leads to increased glucose-induced membrane depolarization [67].

GLP-1 has a physiological circadian secretory rhythm regulated by the core clock gene *BMAL1*, as well as the intestinal environment, with a peak at 2 pm [68]. The homeostasis of this rhythm plays a crucial role in connecting intestinal endocrine cells and pancreatic β-cells [69].

Leptin was shown to enhance GLP-1 secretion in vivo in rodents and in vitro from rodent and human enteroendocrine L cells [70]. Leptin receptors are present in endocrine L cells and neurons secreting GLP-1 [70]. When the circadian rhythm is disrupted, it results in REV-ERBα-mediated leptin resistance [71]. Leptin resistance is found to be associated with decreases in both basal and nutrient-stimulated GLP-1 secretion [70]. GLP-1 receptor agonists (RAs) and dipeptidyl peptidase-4 inhibitors (DPP-4is), which enhance GLP-1 function, may be used to compensate for the GLP-1 deficiency due to circadian rhythm dysfunction and obesity [66,72].

##### RAAS

Plasma renin activity gradually decreases during the day, reaching its nadir at 4 pm, followed by a gradual increase overnight, and peaking at 8 am [73,74]. Plasma angiotensin II (Ang-II) activity also exhibits a diurnal variation, with the highest and lowest values detected at 8 am and 8 pm, respectively [73,74]. This coincides with higher blood pressure (BP) rates in the morning [73,74]. Due to decreased plasma renin activity and sympathetic tone at night, healthy individuals experience a 10–20% decrease in BP at night [73,74]. Obesity correlates with increased activity of angiotensin-converting enzyme (ACE) in white adipose tissue, which results in higher plasma concentrations of Ang-II and loss of its diurnal rhythm [73,74]. A lack of night-time rest is followed by disrupted renin activity, given that plasma renin activity follows the pattern of sleep. This contributes to hypertension development [73]. An absent or blunted night-time decrease in BP (termed “non-dippers”), is associated with increased CV morbidity [73].

PPARγ has its implications in RAAS-mediated hypertension [75]. PPARγ activation lowers BP in humans by antagonizing the RAAS [75]. Activation of PPARγ might antagonize the RAAS, by inhibiting expression of Ang-II and the angiotensin 1 receptor (AT_1_R) in vascular smooth muscle cells. PPARγ may also regulate expression of the renin and angiotensinogen (*AGT)* genes [75].

Increased AT_1_R expression, which may cause hypertension through ROS and inflammation, is brought on by PPARγ mutations [75]. In cells from the affected patients, the surge in renin and AGT boosts Ang-II synthesis, which results in a feed-forward mechanism that may further amplify AT_1_R signaling [75]. The PPARγ mutation-induced increase in ROS, nfKB, and IL-6 is blunted by treatment of the patient’s fibroblasts with rosiglitazone, which presumably activates wild-type PPARγ [75]. As a result, TZD activation of wild-type PPARγ could restore a normal phenotype at the cellular level [75]. 

## 3. Circadian Rhythm Disruption

The most common disruptors of circadian rhythm in humans are lack of sleep, shift work, inadequate food timing, increased nocturnal activity, and use of electronic devices before bedtime [36,76]. Daylight saving time has also been shown to contribute to circadian rhythm disruption and, consequently, to MetS and CVD [77,78]. Improper timing of environmental cues, due to increased night-time activity and disrupted feeding patterns, shifts the phase of circadian rhythms by many hours in peripheral clocks, such as the liver, adipose tissue, and muscles, without significant effects on the SCN [8]. This internal desynchrony in the organism, with central and peripheral clocks out of phase, has been associated with metabolic disorders [8]. Implications of circadian rhythm disruption on all five components of MetS that have been studied so far are summarized in the section below and in Table 1.

## 4. Treatment of MetS in Alignment with Circadian Rhythm

**Figure 2:**
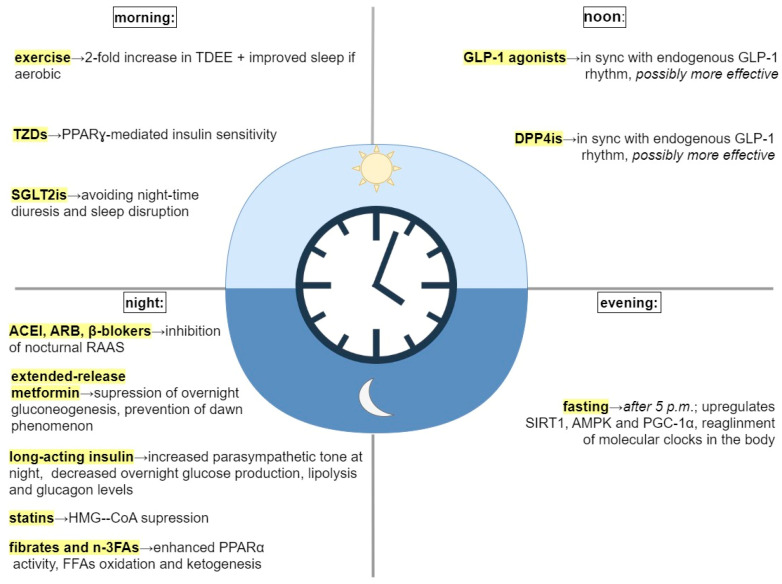
Treatment interventions and their timing. Abbreviations: TDEE, total daily energy expenditure; TZDs, thiazolidinediones; PPARγ, proliferator−activated receptor γ; SGLT2is, sodium−glucose transport protein 2 inhibitors; GLP−1, glucagon−like peptid−1; DPP4is, dipeptidyl peptidase−4; SIRT1, sirtuin1; AMPK, adenosine monophosphate (AMP) activated protein kinase; PGC−1α, peroxisome proliferator-activated receptor−gamma coactivator 1α; ACEI, angiotensin−converting enzyme inhibitors; ARB, angiotensin receptor blocker; RAAS, renin−angiotensin−aldosterone system; HMG−CoA, β−hydroxy−3-methylglutaryl−CoA; n−3FAs, omega−3 fatty acids; PPARα, proliferator-activated receptor α; FFAs, free fatty acids.

Restoration of disrupted circadian rhythm could improve routine treatment in patients with MetS. In accordance with the most recent findings of studies on chronotherapy, the proper timing of routine activities, such as feeding, exercise, and medication, is just as important as lifestyle and pharmacological interventions themselves [9,36,41,61,66,81,83,87,88,89,90,91] (Table 2, Figure 2).

The new information was assessed in light of the current Standards of Medical Care in Diabetes by the American Heart Association used in prevention and delay of T2DM and associated comorbidities [99].

### 4.1. Feeding According to the Biological Clock as a Circadian Rhythm Realignment Strategy

TRF is a dietary strategy that consolidates all calorie intake into a daily window of 6 to 10 h [61,63,100,101]. Many studies demonstrate that TRF is a strong stimulus, capable of resetting both central and peripheral clocks [36,61,63,100,101]. Time restriction of food availability promotes complex changes in the phase and amplitude of clock-controlled gene expression. Resynchronization of the rhythms happens during fasting [9,36]. Fasting promotes activation of SIRT1, AMPK, and PGC-1α-signaling pathways [9]. This results in a phase advance and beginning of the new circadian cycle at the same time throughout the body [9]. Increased AMPK activity promotes FFA oxidation and inhibits ACC, one of the enzymes involved in fat storage [9]. SIRT1 improves insulin secretion and sensitivity, and suppresses inflammatory cytokine expression, which is an important component of MetS [38]. PGC-1α enhances hepatic fatty acid oxidation, which has a positive effect on TG levels [43].

Parr et al. compared the effects of TRF (feeding window of 7 h/day, first meal consumed at 10 am, last meal of the day at 5 pm) vs. extended feeding (EXF; feeding window of 14 h/day, first meal at 07 am, last meal at 9 pm) on glucose and insulin levels, in overweight men [92]. TRF improved nocturnal and postprandial blood glucose control [92]. The 24-h total area under the curve (AUC total) for venous glucose appeared (*p* = 0.09) to be lower for TRF compared with EXF (5.5 9.0 mmol/L/h), which was primarily caused by nocturnal (sleep) glucose AUC being lower in the TRF condition (−4.2 ± 5.8 mmol/L/h, *p* = 0.04) [92]. AUC total for venous insulin levels was generally lower in the TRF condition compared to EXF, although not substantially (*p* = 0.11; 114 197 mIU/mL/h) [92].

Considering the benefits of TRF, a question that imposes itself is: does the exact timing of the eating window matter? An early TRF, where the food intake occurs between 8 am and 6 pm, facilitates weight loss and appetite reduction in people with increased body mass index (BMI), and also has beneficial effects on insulin sensitivity, postprandial glycemia, lipid levels, and BP [62,64,87,102]. On the other hand, restricting food intake to the late afternoon or evening (first meal after 12 pm) does not affect or even worsens these parameters [62,64,87,102]. In addition, late TRF results in inconsistent weight loss, ranging from a slight to no discernible change in weight and whole-body fat mass [62,64,87,102]. Considering all of these facts, TRF with food consumption limited to the early active phase (short feeding window of 7 h and first meal in the morning) could be an extremely beneficial treatment approach in patients with MetS [102].

Furthermore, Jakubowitz et al., in their study, compared two isocaloric weight-loss groups and found that the group that received a larger breakfast and a smaller dinner showed higher improvement in metabolic indicators (body weight and BMI, WC, serum glucose, insulin, ghrelin, and lipids), compared with the group who received a smaller breakfast and a larger dinner [103]. The larger breakfast group showed a 2.5-fold greater weight loss and greater reduction in WC [103]. Fasting glucose, insulin and ghrelin also decreased to a greater extent in the larger breakfast group [103]. After 12 weeks, mean serum TG concentrations decreased by 33.6% in the larger breakfast group, but increased by 14.6% in the larger dinner group [103]. HDL cholesterol slightly but significantly increased only in the group that received a larger breakfast [103].

A prospective study conducted by Timlin et al. demonstrated that the frequency of breakfast among adolescents was inversely associated with BMI in a dose-response manner [104]. Energy, carbohydrate, and fiber consumption were greater among breakfast eaters, but saturated fat consumption was lower [104]. Those who regularly ate breakfast appeared to be substantially more physically active than those who skipped it [104].

Wilkinson et al. assessed whether TRF can act synergistically with pharmacotherapy in a small cohort of patients with MetS who had an unrestricted eating pattern pre-trial [105]. In this study, TRF (eating window 10 h/day for 12 weeks) showed an additive effect to pharmacotherapy (statins and/or anti-hypertensive treatment), by reducing WC, as well as whole-body and visceral fat, lowering BP, and decreasing glycosylated hemoglobin and serum lipids [105]. After 12 weeks of TRF, the mean body weight and BMI reduction were both 3% [105]. The decrease in body weight accompanied desirable reductions in body fat (3%) [105] The mean WC reduction was 4% [105]. The mean systolic and diastolic BP reduction were 4% and 8%, respectively [105]. After 12 weeks, glycosylated hemoglobin dropped 1–3.7% from baseline [105].

These results indicate that TRF should be added to the standard medical practice to treat MetS.

Intermittent fasting (IF) is an eating pattern which consists of changing regularly between periods of eating and fasting [9,106]. IF encompasses different fasting regimens, among which the most popular are alternate day fasting (ADF), which involves alternating 24 h of minimal food intake with 24 h of unrestricted intake, and the 5:2 diet, which includes regular food intake 5 days a week and 2 days fasting [106]. Fasting activates multiple nutrient-responsive pathways, including the insulin/insulin-like growth factor (IGF-1) pathway and adenosine monophosphate-activated protein kinase (AMPK) [107]. It upregulates SIRT1 expression [107]. Hepatic lipid droplets are targeted during intermittent fasting, which ultimately leads to TG hydrolysis via lysosomal acid lipase [22], mediated by PPARα [108].

ADF has been shown to be effective in improving metabolic indicators in non-obese subjects [109]. Following a 12-week ADF, subjects demonstrated a loss of 6.5 kg ± 1.0% in body weight and a reduction of 3.6 ±0.7 kg in fat mass, compared to controls [109]. TG concentrations decreased by 20 ± 8%, and LDL particle size increased in the ADF group relative to controls [109]. CRP decreased (13 ± 17%, *p*  <  0.05) in the ADF group relative to controls at week 12 [109]. Plasma adiponectin increased (6 ± 10%, *p*  <  0.01), while leptin decreased (40 ± 7%, *p*  <  0.05), in the ADF group versus controls by the end of the study [109].

ADF has a positive effect on obese subjects as well [110]. After 10 weeks of ADF in obese individuals, body weight decreased by 5.6 ± 1.0 kg, percentage of body fat decreased by 3 ± 2%, total cholesterol, LDL, and TG concentrations decreased by 21 ± 4%, 25 ± 10%, and 32 ± 6%, respectively [110]. HDL cholesterol remained unchanged. Systolic blood pressure decreased from 124 ± 5 to 116 ± 3 mm Hg [110].

Tripolt et al. examined glucose metabolism and metabolomics profiles after 12 h and 36 h fasting in non-obese and obese participants and people with T2DM [111]. Fasting glucose was not significantly changed from baseline, but fasting insulin was significantly lower in both men and women (*p* < 0.001) [111]. Fasting β-hydroxybutyrate and FFA concentrations were higher by the end of the study in both male and female subjects [111]. HDL was elevated from baseline in the women only (*p* < 0.001), and TGs were significantly reduced from baseline in the men only (*p* < 0.05) [111].

Kang et al. demonstrated that an IF 5:2 regimen produced superior weight loss (7.9 ± 5.0 kg), compared to daily caloric restriction (4.7 ± 3.4 kg), during a 12-week study on Chinese overweight and obese patients [112].

Despite the fact that it has been shown as a beneficial treatment intervention for weight loss and the improvement of metabolic indicators, it is questionable whether IF could be applicable on a day-to-day basis, especially in elderly populations and in patients with T2DM. The common adverse effects of IF include dizziness, headache, nausea, irritability, hypoglycemia, and temporary sleep disturbances [106,112,113]. Nevertheless, in studies with IF, adverse effects were less common and less severe over time, suggesting that the body takes some time to adapt [106,112,113].

### 4.2. Exercise around the Clock

Current Standards of Medical Care in Diabetes by the American Heart Association recognize that overweight and obese people with an increased risk for developing T2DM should enroll into programs for lifestyle behavior change [99]. They should introduce moderate-intensity aerobic activity, such as a brisk walk 150 min/week, and eat less fat and fewer calories with a goal to lose 7% of their initial body weight (grade A evidence) [99]. Exercise timing has a bimodal on the circadian clock [42]. Firstly, it acts as a time cue, crucial in the realignment of disrupted central and peripheral circadian clocks [42].

Many proxies of circadian rhythm have been reported to be altered by exercise, including hormone secretion (e.g., melatonin, cortisol, and thyroid-stimulating hormone [TSH]) and physiological parameters (e.g., body temperature, BP) [114,115]. For instance, melatonin phase delays are associated with exercise in the evening or overnight [115].

Secondly, exercise’s metabolic outputs might depend on its daily timing [42].

Sato et al. examined how the timing of exercise impacts local tissue and systemic metabolism in mice [35]. Exercise or control sham exercise was performed for 1 h on a treadmill at either early light/rest phase (ZT3) or early dark/active phase (ZT15), followed by the detection of metabolites such as ketones, amino acids (AAs), lipids in WAT, muscle, and serum [35]. Exercise at ZT15, i.e., in the early active phase, increased the levels of beta-hydroxybutyrate (BHB) and urea, indicating a greater dependence on FFA oxidation and enhanced buffering against metabolic stress [35]. Acyl-carnitine levels increased more upon ZT15 exercise in both muscle and serum, which suggests that exercise at ZT15 activates FFA oxidation in muscle and increases the demand for energy from non-glycolytic sources [35]. These findings are confirmed by gene expression profiling, which found that muscle genes involved in FFA oxidation, including Pparδ, were specifically upregulated following exercise at ZT15 [35]. Exercise at ZT15 also boosted muscle AMP levels and activated AMPK [35]. Activated AMPK phosphorylates and destabilizes circadian transcriptional repressors CRY1/2 [35]. This allows the de-repression of *Bmal1:Clock* targets, which results in the reprogramming of the circadian and gluconeogenic genes [35]. Thus, exercise may reset misaligned muscle clocks if timed appropriately, i.e., in the early-active phase [35].

Asher et al. studied the variation in exercise capacity of wild-type mice between two distinct time points during their active phase, namely 2 h and 10 h within the dark phase [116]. Exercise had a different impact on gene expression in the Early group compared to the Late group [116]. Insulin-signaling pathways and glucose metabolism were enriched specifically in the Early group. Moreover, PPAR was upregulated only in the Early group [116]. Following exercise, the Early group displayed a more pronounced decline in lipids and amino acids, compared to the Late group [116].

Asher at al. also detected higher oxygen consumption in humans during exercise in the early phase (8 am) vs. late phase (6 pm) [116]. Evening exercise is associated with greater exercise capacity and endurance, which is related to a greater reliance on carbohydrates and higher body temperature in the evening [116]. Carbohydrates require less oxygen per amount of ATP produced, which may contribute to the lower oxygen consumption and lower glycemia upon evening exercise [116]. On the other hand, muscle cells seem to be more effective at FFA oxidation in the morning, potentially leading to greater fat loss [116]. Therefore, morning exercise could be an effective strategy for people with obesity and/or T2DM [116].

In a study by Creasy at al., during A 15-week exercise protocol, morning exercise (6–10 am) resulted in a >2-fold increase in total body energy expenditure compared with evening exercise (5–7 pm) [93]

According to research by Willis et al., obese young adults lost significantly more weight over the course of 10 months of high-intensity, supervised aerobic exercise (2000–3000 kcal/week) in the morning (−7.2 ± 1.2%; *p* < 0.001) than they did in the evening (−2.1 ± 1.0%; *p* < 0.001) [65].

In a study performed by Tokuyama et al., 24 h FFA oxidation was the highest in participants who exercised in the morning before breakfast, compared with those who exercised in the afternoon, evening, and sedentary controls [117]. Transient carbohydrate deficits, i.e., glycogen depletion observed after morning exercise, may have contributed to increased 24 h fat oxidation [117].

Several studies demonstrate how aerobic exercise supports circadian alignment to optimize health outcomes [94,95,96]. Van Someren and colleagues found that 3 months of consistent aerobic exercise, performed three times a week, counteracted age-related disruption of the circadian clock and improved sleep quality in otherwise healthy older men (73 ± 2 years) [94]. A significant correlation was found between maximal aerobic fitness and reduced circadian variability after the intervention, an important finding given the association between aerobic capacity and all-cause mortality [95,96].

### 4.3. Circadian Medication

The availability of binding sites for medications used in the treatment of MetS shows circadian oscillations, and it is established that effectiveness, as well as toxicity, of these drugs varies depending on the specific time of the day [118].

#### 4.3.1. Antihypertensives

It is no wonder that chronotherapy has its roots in the treatment of hypertension, considering the fact that day–night variations in BP are among the best-known circadian rhythms of physiology [83,88,89,90,119]. ACE inhibitors (ACEIs) and angiotensin receptor blockers (ARBs), are proven to be more effective when administered in the evening, compared with morning-dosed controls [83,90,119]. Their better efficacy at night can be explained by the inhibition of nocturnal RAAS activation [74].

Numerous studies support this theory [83,88,89,90,119]. Kuroda et al. demonstrated that the long-acting ACEI trandolapril was a safer and more effective after bedtime administration, compared with morning administration [88]. Hermida et al. demonstrated that bedtime spirapril administration was more efficient in BP control during both nocturnal sleep and daytime activity, compared with morning administration [119]. In another study by Hermida et al., the reduction in the 48 h mean values for systolic and diastolic BP was greatest when a valsartan/amlodipine combination was ingested at bedtime [89]. Moreover, in a study on non-dipper patients with essential hypertension (grade 1–2), valsartan administration at bedtime, as opposed to upon awakening, showed improved efficacy in BP control during the nocturnal resting hours [83]. In this study, 75% of the patients in this group reverted to dippers, there was a significant increase in the percentage of patients with controlled BP over 24 h, and there was a reduction in urinary albumin excretion [83].

There are no papers on the timing of other groups of antihypertensives, to the best of our knowledge.

#### 4.3.2. Antihyperglycemic Medications

##### Metformin

Metformin is a widely-used treatment option for “prediabetes”, i.e., IR, in MetS, and its mechanism of action has been linked to the activation of AMPK, a positive-loop regulator of the circadian rhythm [41]. AMPK promotes the degradation of PER2 through the activation of CK1ε, which leads to the phase advance of circadian clocks in the peripheral tissues [41]. Metformin could also have beneficial effects against oxidative injury, given that it activates PGC-1α via AMPK induction, to restore the mitochondrial network and to counteract ROS generation [44].

The effects of metformin are tightly linked to circadian rhythm, which emphasizes the importance of proper timing of metformin administration [41]. Extended-release metformin should be taken once a day at night, with dinner. Bedtime administration is proven to be the most effective in blood glucose regulation, given that, by targeting clock genes, metformin suppresses overnight gluconeogenesis and prevents morning hyperglycemia [41].

##### Imeglimin

Imeglimin (IMEG), the first of the group of oral tetrahydrotriazine compounds, has also been found to activate AMPK, but to a lesser extent, compared to metformin [120]. A recent study by Hozumi and colleagues found that IMEG reduced the ATP/ADP ratio in primary cultured hepatocytes, which was the most probable trigger for AMPK activation [120]. The measured AMPK rates, however, were lower than those induced by metformin [120]. Although IMEG exerts similar effects to those of metformin on AMPK activity, it is yet to be determined whether it is potent enough to phase-shift the circadian rhythms [120]. IMEG is currently administered twice daily, in the morning and evening [121], but further studies are needed to determine whether IMEG’s effects depend on the time of day it is administered.

##### PPARγ Agonists

TZDs, as full agonists of PPARγ, have been shown to improve insulin sensitivity [97]. They affect catabolism of TGs to a lesser extent [97]. They could also have beneficial effects on the CV system, given that PPARγ plays essential role in maintaining the circadian rhythms of BP and heart rate [97].

A recent study in mice showed that pioglitazone can resynchronize clock genes and inflammation-related genes in the mouse liver that have been disrupted by reverse feeding, i.e., feeding during the rest phase [91]. This resynchronization resulted in decreased hyperglycemia, hypercholesterolemia, and transaminase activity, as well as decreased IL-6 in liver tissue [91]. It is yet to be determined whether the same results can be achieved in humans.

PPARγ expression in adipose tissue and skeletal muscle peaks at the beginning of the active phase [20]. This means that morning administration of TZDs could be more efficient in IR treatment. Further research is needed to provide more evidence in support of this hypothesis.

##### GLP-1 RAs

GLP-1 RAs offer pharmaceutical levels of GLP-1, which lower blood sugar levels and body weight, by enhancing glucose-dependent insulin secretion, decreasing glucagon secretion, prolonging gastric emptying, and inducing satiety [69].

Although, to the best of our knowledge, there are still no studies related to this subject, it is possible that GLP-1 RAs, such as liraglutide and lixisenatide, could have a stronger beneficial effect if administered in sync with endogenous GLP-1 rhythms.

##### DPP-4is

DPP-4is are antihyperglycemic drugs which prevent the inactivation of GLP-1, thereby increasing its levels and potentiating its action [72].

Although, to the best of our knowledge, there are still no studies related to this subject, DPP-4is could have a stronger beneficial effect if administered in sync with endogenous GLP-1 rhythms [69].

##### SGLT2 Inhibitors

Sodium-glucose cotransporter-2 inhibitors (SGLT2is) inhibit renal glucose reabsorption by blocking the SGLT2 cotransporters in the proximal tubules and causing glucosuria [122]. The accompanying sodium excretion explains their additional effect on lowering BP [122]. Their implication with regard to the circadian pressure has been shown, and emerging data suggest that SGLT2is not only decrease BP, but also improve its disrupted circadian rhythm [98]. Nevertheless, there is no significant difference in the effectiveness of SGLT2is between morning and evening administration [123]. Evening administration might be less favorable because these medications increase urinary volume and disrupt night-time sleep with frequent bathroom visits, which might negatively affect circadian balance [98].

##### Insulin

Porcellati and colleagues observed significant differences in the pharmacokinetics and pharmacodynamics of basal insulin glargine after morning vs. evening administration [124]. With morning or evening glargine dosing, total insulin activity on glucose metabolism was similar [124]. Evening glargine administration, however, consistently reduced nocturnal endogenous glucose production, lipolysis, and glucagon concentration [124]. Thus, compared to morning glargine dosing, targeting fasting euglycemia with evening glargine dosing seems more convenient [124].

A study by Takeshita et al. compared the effects of two different insulin regimens—basal (insulin glargine) versus bolus insulin (glulisine)—on metabolic and cardiovascular autonomic function in Japanese participants with T2DM [125]. Insulin glargine, but not insulin glulisine, increased parasympathetic tone during night-time and decreased sympathetic nerve activity at dawn [125]. These findings shed light on the previously unrecognized role of night-time basal insulin supplementation on sympatho-vagal circadian rhythm in T2DM [125].

#### 4.3.3. Hypolipidemic Agents

##### Statins

Statins, 3-hydroxy-3-methylglutaryl-coenzyme A (HMG-CoA) reductase inhibitors, are considered a standard therapy for many types of dyslipidemia. Circadian-regulated statin administration was established decades ago [81]. They are generally administered in the evening, because HMG-CoA reductase-regulated cholesterol biosynthesis peaks during the night [81]. Awad and colleagues found that short-acting statins taken in the evening were significantly more effective in lowering LDL-C and total cholesterol, consequently reducing CVD risk, than those taken in the morning [81]. Furthermore, in a randomized control trial by Wallace et al., simvastatin was found to be significantly more effective in lowering LDL-C and total cholesterol when taken at night [126].

##### PPARα Agonists

PPARα agonists (fibrates) and omega-3 fatty acids are powerful TG-lowering agents. They affect TG catabolism by promoting β-oxidation and raising the levels of HDL [127]. They are also powerful stimulators of ketogenesis [128]. One of their downsides is the contribution to gallstone formation [129]. They inhibit bile acid synthesis, via PPARα-mediated downregulation of cholesterol 7α-hydroxylase and sterol 27-hydroxylase [129]. It is known that these rate-limiting enzymes exhibit diurnal rhythmicity, with the highest activity at noon [130]. The current recommendation is to take fibrates with a meal, once a day [131]. If fibrates were to be taken at night, they would enhance physiological PPARα night-time activity and physiological TG catabolism [86,132]. On the contrary, when taken during the day, they would activate PPARα at the wrong time and place, which would result in the suppression of bile acid synthesis and, thus, a higher probability of gallstone formation [86,132].

##### PPARβ/δ Agonists

In addition to the insulin-sensitizing and antihyperglycemic effects of TZDs, PPARβ/δ agonists, such as elafibranor and telmisartan, achieve TG-lowering and HDL-raising effects [91]. They also have potential for treating NAFLD, which is closely related to MetS [91]. Telmisartan is also ARB and is widely used in the treatment of hypertension [90]. Its antihypertensive effect is significantly better with bedtime dosing [90]. Telmisartan could potentially be an optimal medication for patients with MetS, considering its beneficial effects on insulin sensitivity, lipid levels, and hypertension.

To the best of our knowledge, there is no data on the timing of administration of other hypolipidemic agents, such as proprotein convertase subtilisin/kexin type 9 (PCSK9) inhibitors and ezetimibe.

## 5. Conclusions

Recent advances in preclinical studies show that the timing of lifestyle changes is important and should be considered when managing patients with MetS. Not only are the quantity and quality of food and exercise important, their timing is also extremely important.

Timing is essential in the pharmacological treatment of MetS as well. This fact is not emphasized enough in the current standards of care for people with MetS. Circadian rhythms are often seen as regulators of sleep–wake patterns, but their importance is becoming increasingly evident in regulation of the functions of many systems in the body. More studies focused on synchronizing the timing of the food intake with the circadian clock are needed, in order to reach a strong consensus on recommendations regarding this novel strategy for weight loss, and in order to decrease the incidence of MetS in the general population. We can anticipate future changes to evidence-based recommendations, based on the chronobiology-based exercise treatment approach.

## 6. Literature Review

In this review, we summarized English-language publications from PubMed, as well as the Cochrane Central Register of Controlled Trials. We searched the literature from 1 January 2009 to 1 February 2023, using Medical Subject Headings (MeSH) keywords: metabolic syndrome, diabetes mellitus, circadian rhythm, circadian clocks, diet, exercise, and chronotherapy. Clinical trials related to the importance of the circadian rhythm in the regulation of metabolic processes were included. English-language articles related to the topic were considered if they discussed one of the issues of interest and were peer reviewed.

## Figures and Tables

**Table 1:** MetS as a consequence of circadian rhythm disruption.

IR	Central Obesity	Dyslipidemia	Hypertension
Disrupted SIRT1 expression results in: diminished mitochondrial regeneration capacity overproduction of ROS and proinflammatory cytokines [38,39]. NADH accumulation in the cytoplasm [1] Inhibition of FFA oxidation–FFA levels in cytoplasm rise and inhibit GLUT4 translocation [1,38].	Phase delay leads to REV-ERB-dependent leptin resistance in SCN [15,71] and decrease in POMC Low POMC is associated with increased appetite, reduced energy expenditure, and increased fat deposition [49].	Expression of genes involved in lipid and cholesterol metabolism, such as HMG-CoA reductase and SREBF1 are attenuated or abolished in PPAR*α*-knockout mice [20].	Obesity correlates with increased activity of ACE in white adipose tissue, which results in higher plasma concentrations of angiotensin II and loss of its diurnal rhythm [74].
Dampened PPARγ expression [31]: Loss of insulin receptor phosphorylation Decrease in PI3K activity and cellular glucose uptake [32].	There is a significant difference between circadian rhythm of adiponectin and its receptors in subcutaneous versus visceral fat [50,79,80,81]. Larger amount of visceral white adipose tissue is associated with dampened rhythm of adiponectin, and IR [50,79,80,81].	The hepatic PPAR*α*-knockout mice show significantly increased levels of LDL subfraction L5 in response to high-fat feeding [82].	Non-dipping hypertension (i.e., absence of a physiological 10–20% decrease in BP at night) is associated with activation of the RAAS, increased risk of chronic kidney disease, and adverse CV events [74,83].
Adiponectin rhythm delay, associated with both circadian disruption and central obesity [50,84,85].	Changes in adipocytes’ metabolism, leading to down-regulation of PPARγ [31]. PPARγ repression leads to reduced histone acetylation and methylation, thereby repressing *BMAL1* transcription and expression in adipocytes [31].	Proinflammatory macrophage markers TNF and iNOS are increased in the liver of the PPAR*α* knockout mice compared to controls [86].	

Abbreviations: IR, insulin resistance; SIRT1, sirtuin 1; ROS, reactive oxygen species; NADH, nicotinamide adenine dinucleotide (NAD) + hydrogen (H); FFA(s), free fatty acid(s), GLUT4, glucose transporter type 4; PPARγ, peroxisome proliferator-activated receptor γ; PI3K, phosphatidylinositol-3-kinases; REV-ERB, reverse-strand avian erythroblastic leukemia (ERBA) oncogene receptor; SCN-suprachiasmatic nucleus; POMC, proopiomelanocortin; *BMAL1*, gene-encoding Brain and Muscle Arnt-Like protein 1; HMG-CoA, 3-hydroxy-3-methylglutaryl-coenzyme A; SREBF1, sterol regulatory element binding transcription factor 1; PPAR*α*, peroxisome proliferator-activated receptor *α;* LDL, low density lipoprotein; TNF, tumor necrosis factor; iNOS, inducible nitric oxide synthase; ACE, angiotensin-converting enzyme; BP, blood pressure; RAAS, renin–angiotensin–aldosterone system; CV, cardiovascular.

**Table 2:** Summary of treatment recommendations for patients with MetS, with regards to the circadian clock.

Intervention	Recommended Timing	Explanation
Food consumption (without specific diet plan)		
Time-restricted feeding (TRF)	Early TRF- food intake between 8:00 and 17:00	Fasting in the late-active and rest phase upregulates SIRT1, AMPK, and PGC-1α, and causes a phase-advance and the alignment of molecular clocks throughout the body [9,92].
Exercise		
Aerobic exercise	Morning	Morning exercise is associated with a 2-fold increase in total body energy expenditure and more extensive FFA oxidation compared to evening exercise [93]. Aerobic exercise restores disrupted circadian rhythm and improves sleep [94,95,96].
Medication		
Antihypertensive medication (e.g., ACEI, ARB)	Bedtime	Better efficacy of ACEI, ARB, and β-blockers at night can be explained by the inhibition of nocturnal RAAS [74,83,88,90].
Extended-release metformin	Bedtime, with dinner	By targeting clock genes, suppresses overnight gluconeogenesis and prevents morning hyperglycemia (i.e., dawn phenomenon) [41].
Thiazolidinediones (PPARγ agonists)	Morning	Boost PPARγ-mediated insulin sensitivity in AM [91,97].
GLP-1 agonists (liraglutide, lixisenatid)	Authors’ suggestion: noon	GLP-1 activity peaks at 14:00 [66,69]. It is possible that GLP-1 analogs could be more effective if administered in sync with endogenous GLP-1 rhythms [66].
DPP4is	Authors’ suggestion: noon	It is possible that DPP4is could be more effective if administered in sync with endogenous GLP-1 rhythms [69,72].
SGLT2is	Authors’ suggestion: morning	No significant difference in effectiveness of SGLT2is in morning vs. evening administration [98]. Evening administration might negatively affect circadian rhythm by increasing diuresis and disrupting night-time sleep [98].
Insulin (long acting)	Bedtime	Evening glargine decreases nocturnal endogenous glucose production, lipolysis, and glucagon concentration. It increases parasympathetic tone during night-time and decreases sympathetic nerve activity at dawn, which can improve treatment of morning hyperglycemia [46,52].
Statins (esp. short-acting)	Bedtime	Statins suppress HMG-CoA reductase-regulated cholesterol biosynthesis, which peaks at night [81].
Fibrates and omega-3-fatty acids (PPARα agonists)	Bedtime, with dinner	At night, they enhance PPARα physiological activity—β-oxidation and ketogenesis. Bedtime administration could reduce side effects—gallstone formation [20,86].

Abbreviations: TRF, time-restricted feeding; SIRT1, sirtuin1; AMPK, adenosine monophosphate (AMP) activated protein kinase; PGC-1α, peroxisome proliferator-activated receptor-gamma coactivator 1α, ACEI, angiotensin-converting enzyme inhibitors; ARB, angiotensin receptor blocker; RAAS, renin–angiotensin–aldosterone system; PPARγ, proliferator-activated receptor γ; HMG-CoA, β-hydroxy-3-methylglutaryl-CoA; PPARα, proliferator-activated receptor α; GLP-1, glucagon-like peptid-1; SGLT2is, sodium-glucose transport protein 2 inhibitors; DPP4is, dipeptidyl peptidase-4.

## Data Availability

Not applicable.
